# Case of Schirrus Lung

**Published:** 1852-09

**Authors:** Judson Bowen

**Affiliations:** Pampas, Illinois


ART. VII.—*Case of Schirrus Lung.* By JUDSON BOWEN, M. D., of Pampas, Illinois.

Mr. L. W. W., aged 42, applied to me in April, 1850, complaining of a severe pain in his right side and shoulder, cough, dyspnœa, loss of appetite, &c. The previous history of the case, so far as I was able to learn, is briefly this:—About five years previous to the time he applied to me, a small tumor appeared on his chest, directly over the sternum, which continued slowly to increase in size until Feb., 1847, when it was taken out by Doct. Richards, of St. Charles. The wound healed and the patient appeared quite well, until a short time before he applied to me, at which time another tumor had appeared upon his side, over the middle of his right lung, about mid-way between the spine and sternum. Believing the case to be one of importance, I advised the patient to obtain the counsel of Dr. Hard, of Aurora, who pronounced the tumor true schirrus, and advised its removal. It was removed in May following, and, with Dr. Hard's advice, the patient wa, placed under a tonic and expectorant treatment, consisting principally of iodine of iron, mineral acids, and balsam copaive.

The patient's debility continued; with loss of appetite, great difficulty of breathing, and emaciation, until August 10th, f llowing, when he expired. A number of tumors of the same nature had appeared upon various parts of the body and limbs previous to his death. I had diagnosed from the first, as also had Dr. Hard, the principal cause of his lung difficulty to be malignant. Dr. Hopkins was present with myself at the post mortem, which from unavoidable circumstances was incomplete, the lungs only being hastily examined. Opposite the middle and posterior portion of the middle lobe of the right lung, was found an enormous deposit of schirrus matter, consisting principally of smaller tumors conglomerated together, and adhering to the lung and pleura; the lower lobe of the right lung was comparatively healthy; the middle and upper lobes considerably diseased. The left lung was healthy, excepting the upper lobe, which was found slightly tuberculous.

